# Seeking the patient perspective

**DOI:** 10.1111/hex.12801

**Published:** 2018-06

**Authors:** Carolyn A. Chew‐Graham

**Affiliations:** ^1^ Research Institute Primary Care and Health Sciences Faculty of Medicine and Health Sciences Keele University

The concept of Patient and Public Involvement and Engagement (PPIE) in health service provision and research has been around since the World Health Organisation published the Alma‐Ata Declaration.^1^ In the fourth declaration of this document, it was explicitly stated citizens' both duty and right to participate in health care implementation and planning.^1^ Since then, PPIE has developed, with governments worldwide developing policies and amendments in their health legislations, to include the involvement of the public in health research and service provision.^2^, ^3^

The emergence of PPIE came after social movements and public demand of involving the public in health services that will be used by and offered to all. The complexity of meeting the diverse health needs of the population can be mitigated by having the public's input on priorities needed to be dealt with, as they will be the consumers. The “public” is not limited to *patients*, but is also used to refer to carers, potential patients, and any other organization that represents people who use health services.^3^ Much literature has explored the impact of PPIE in health services,^4^, ^5^ and there is growing evidence of the impact PPIE has in health research.^6^ PPIE is particularly beneficial to service users and the public when performed beyond a tokenistic approach, despite its monetary and resource costs.^5^

Examples of benefits of including Patient and Public Involvement and Engagement in research can include identifying the important research questions, ensuring participant burden is minimized, supporting analysis and providing advice on dissemination, particularly to lay audiences.^7^

Mental health is one area with strong evidence for PPIE in research.^8^ Evidence suggests that involving the public in mental health research enhances the quality of research^9^, ^10^, ^11^ and that PPIE can not only benefit service users, but may have a positive effect on researchers. Thus, two systematic reviews report that researchers involving PPIE in their work were positively influenced by challenging their attitudes and beliefs towards mental health, as well as enjoying working in partnership with people, and becoming empathetic towards service users.^12^, ^13^

In this edition of HEX, we feature two manuscripts which demonstrate the value of seeking stakeholders' opinions and perspectives about mental health services. Brooks and colleagues report their qualitative study from which they conclude there is a lack of alignment of care planning activities to the every‐day lives of mental health service users. Care planning was fulfilling an organizational goal, seemingly at the expense of delivering the primary stated purpose of care planning—improving patient care. The value of this study for global mental health policy and to those responsible for the planning and delivery of mental health services is emphasized.

Clarke and colleagues report a qualitative study which suggests a number of strategies for engaging young adults with diabetes in mental health research, and offers offer broad suggestions for health professionals and mental health researchers to support involving young patients with diabetes in research; their conclusions seem to be applicable to all young people seeking health care and sensible suggestions for health service researchers.

Critical analysis of previous research using systematic reviews can be helpful in addressing the patient or service‐user perspective. Sutcliffe and colleagues' systematic review highlights the essential value of patient and public views about the health services they receive, by revealing the mismatch between service users' experiences and perceptions of the critical features of weight management plans (WMPs) and the focus of programme descriptions and evaluations. Similarly, Waldecker's systematic review demonstrates that much is still to be learned about written action plans (WAPs) for children. Whilst WAPs have been advocated for people with long‐term conditions, to guide decision making and support self‐management, this review suggests that there remains uncertainty about how WAPs “work” and what aspects are important for successful implementation.

McKevitt and colleagues describe a study using documentary analysis with the aim of understanding how patients and carers were involved in major system changes (MSCs) to the delivery of acute stroke care in 2 English cities, and what kinds of effects involvement was thought to produce. The value of patient and carer involvement is suggested to lie, not in its contribution to acute service redesign, but in the facilitation and support of delivery of the changes developed by professionals.

One paper in this edition of HEX demonstrates how patients can be involved in the conduct of research. Jørgensen aimed to investigate the impact of involving patient representatives as peer interviewers in a research project on patient empowerment.

Differences were identified between the academic researcher and the peer interviewers in the types of questions they asked and the degree to which personal narrative was used in the interview. Peer interviewers varied in their approach. Research participants were positive about the experience of being interviewed by a peer interviewer. No firm conclusions could be made about impact on outcomes. The authors conclude that, in any study where peer interviewers are utilized, it is important to consider potential benefits alongside relevant ethical considerations, available resources for support of both peer interviewers and interviewees, and the need for training, not only in interview techniques, but also in reflexivity and professional/personal boundary work.

HEX aims to publish work which not only researches perspectives of patients, carers and the public about health services, with the aim of improving care offered, but we encourage authors to report the involvement of patients at all stages of their work—from the research question, design and conduct of research, data generation, data analysis and dissemination, to lay and professional audiences.
